# Identifying Therapies to Combat Epithelial Mesenchymal Plasticity-Associated Chemoresistance to Conventional Breast Cancer Therapies Using An shRNA Library Screen

**DOI:** 10.3390/cancers12051123

**Published:** 2020-04-30

**Authors:** Sugandha Bhatia, Tony Blick, Cletus Pinto, Mark Waltham, James Monkman, Ekaterina Ivanova, Pamela M. Pollock, Shivashankar H. Nagaraj, Adrian P. Wiegmans, Izhak Haviv, Kaylene J. Simpson, Erik W. Thompson

**Affiliations:** 1 Institute of Health and Biomedical Innovation and School of Biomedical Sciences, Queensland University of Technology, Brisbane QLD-4102, Australia; tony.blick@qut.edu.au (T.B.); monkman.james@gmail.com (J.M.); pamela.pollock@qut.edu.au (P.M.P.); shiv.nagaraj@qut.edu.au (S.H.N.); 2 Translational Research Institute, Brisbane QLD-4102, Australia; ekaterina.ivanova@qut.edu.au (E.I.); adrian.wiegmans@qut.edu.au (A.P.W.); 3 Invasion and Metastasis Unit, St. Vincent’s Institute, Melbourne VIC 3065, Australia; cletusp136@gmail.com; 4 Pharmacogenomics Unit, St. Vincent’s Institute, Melbourne VIC 3065, Australia; mark.waltham@monash.edu; 5 Institute of Health and Biomedical Innovation, Cancer & Ageing Research Program, Queensland University of Technology, Brisbane 4102, Australia; 6 Centre for Tumour and Immune Biology (ZTI), Department of Medicine, Philipps University Marburg, Marburg 35043, Germany; 7 Victorian Centre for Functional Genomics, Peter MacCallum Cancer Centre, Melbourne, Victoria 3000, Australia; izhak@aidgenomics.com (I.H.); kaylene.simpson@petermac.org (K.J.S.); 8 Sir Peter MacCallum Department of Oncology, University of Melbourne, Parkville, Victoria 3010, Australia; 9 AID Genomics, Prof. Menahem Plaut 10, Rehovot 7670621, Israel

**Keywords:** chemotherapy resistance, combination chemotherapy, doxorubicin, docetaxel, eribulin, TGFBR, FGFR, MDM, TP53, shRNA library screening

## Abstract

Background: Breast cancer (BC) is a heterogeneous disease for which the commonly used chemotherapeutic agents primarily include the anthracyclines (doxorubicin, epirubicin), microtubule inhibitors (paclitaxel, docetaxel, eribulin), and alkylating agents (cyclophosphamide). While these drugs can be highly effective, metastatic tumours are frequently refractory to treatment or become resistant upon tumour relapse. Methods: We undertook a cell polarity/epithelial mesenchymal plasticity (EMP)-enriched short hairpin RNA (shRNA) screen in MDA-MB-468 breast cancer cells to identify factors underpinning heterogeneous responses to three chemotherapeutic agents used clinically in breast cancer: Doxorubicin, docetaxel, and eribulin. shRNA-transduced cells were treated for 6 weeks with the EC_10_ of each drug, and shRNA representation assessed by deep sequencing. We first identified candidate genes with depleted shRNA, implying that their silencing could promote a response. Using the Broad Institute’s Connectivity Map (CMap), we identified partner inhibitors targeting the identified gene families that may induce cell death in combination with doxorubicin, and tested them with all three drug treatments. Results: In total, 259 shRNAs were depleted with doxorubicin treatment (at *p* < 0.01), 66 with docetaxel, and 25 with eribulin. Twenty-four depleted hairpins overlapped between doxorubicin and docetaxel, and shRNAs for TGFB2, RUNX1, CCDC80, and HYOU1 were depleted across all the three drug treatments. Inhibitors of MDM/TP53, TGFBR, and FGFR were identified by CMap as the top pharmaceutical perturbagens and we validated the combinatorial benefits of the TGFBR inhibitor (SB525334) and MDM inhibitor (RITA) with doxorubicin treatment, and also observed synergy between the inhibitor SB525334 and eribulin in MDA-MB-468 cells. Conclusions: Taken together, a cell polarity/EMP-enriched shRNA library screen identified relevant gene products that could be targeted alongside current chemotherapeutic agents for the treatment of invasive BC.

## 1. Introduction

Many reports have confirmed that preoperative chemotherapy has the ability to modify the genome of tumour cells, can impact the tumour population dynamics, and can induce epithelial–mesenchymal transition (EMT) [1,2,3,4]. While the direct implications of EMP upon metastasis remain controversial, strong supportive evidence has widely implicated EMT and EMT-associated stem-like properties in chemoresistance in vitro and in vivo [3,5,6,7,8,9,10,11,12,13]. The contribution of EMT towards the resistance of cells to undergo apoptosis, anoikis, and cellular senescence likely underpins its role in chemoresistance [14,15,16]. Further studies to comprehend the association between the EMT programme, CSC phenotype, and therapeutic resistance will be a prerequisite to the development of more effective treatment strategies to eliminate the capacity of cancer cells to resist treatments by reprogramming through EMT and/or CSC-like functions. 

The development and improvement of RNA interference (RNAi) libraries, composed of either small interfering RNAs (siRNAs) or vector-based short hairpin RNAs (shRNAs), biomolecules that have the ability to target almost every gene in the genome, has made it conceivable to perform high-throughput screens with the capability of examining distinct phenotypes associated with the loss of function of many genes concurrently [17,18,19,20,21]. The progress in functional RNAi screening technologies has also aided the development of biomarker discoveries against targeted drug-induced cytotoxicity [22,23,24]. Use of an shRNA library, which stably inhibits gene expression and thus protein function on a multi-gene or even genome-wide scale, can model the pharmacological suppression of a target protein, and is consequently an effective approach for the discovery of drug targets. It also provides resources for understanding the relationships underpinning resistance mechanisms to therapeutic modalities. The drop-out viability screen approach can identify ‘synthetic lethality’ genes that, on inhibition via shRNA, sensitise the cell to the drug’s lethal impact [21,25,26,27]. This technique involves treating cancer cells with an effective drug concentration to determine genes for which a loss of function is lethal under those conditions [28]. ‘Hits’ are deduced from the drop-out viability screen in the presence of the drugs, as compared to a control screen that does not include the compound (i.e., significant deviation in the expression of the targeting shRNA in drug treatment versus no drug treatment) [29]. 

The systematic linking of the functional complexity of the genome in cancer cell lines (attenuated using the shRNA library) with pharmacological profiling can pave the way to new cancer treatment strategies that have a reduced risk of developing drug resistance. The drug–genotype associations identified by the drop-out viability method are a proven approach to identifying the sensitivity/resistance patterns in a tumour cell population, providing significant clues to the association of specific drug targets with a cellular phenotype [24]. Following this principle, we conducted a drop-out viability shRNA screen to identify new targets that, when depleted by shRNA, make cancer cells resistant to the therapeutic treatment, and in our screen allow determination of the ‘hits’ that link the EMT and/or CSC programme with drug resistance.

MDA-MB-468 breast cancer cells carrying a lentiviral ‘Polarity Pool’ library of shRNA designed to target genes implicated in cell polarity, epithelial mesenchymal plasticity (EMP), breast cancer stem cells (BCSCs), cell migration, and metastasis were generated, as detailed below. A drop-out viability screen in MDA-MB-468 breast cancer cells was conducted to identify gene targets that mediate resistance in the presence of cytotoxic drugs, including standard-of-care breast cancer chemotherapies (docetaxel, doxorubicin) and the EMT-reversing drug eribulin [30], approved for use with metastatic breast cancer [31,32]. 

Using this approach, we hope to expand our understanding of the relationship between EMP and resistance mechanisms to clinically relevant therapeutic interventions, and also to identify and validate EMP-associated targets (utilising known inhibitory agents) that render the cells resistant or sensitive to the drug therapy. The data generated also has the potential to predict useful biomarkers for treatment response, and elucidate which significant biological pathways are involved in the resistance to specific drug(s).

## 2. Materials and Methods

### 2.1. Cell Line and Growth Conditions

The MDA-MB-468 (‘Basal A’ subgroup [33]), ‘triple-negative’ human breast cancer cell-line was obtained from ATCC (http://www.atcc.org) via the Lombardi Cancer Centre, Georgetown University, Washington, D.C., USA, and cultured in growth medium (Dulbecco’s Modified Eagle Medium (DMEM) supplemented with 10% foetal bovine serum (FBS; GibcoTM, Thermo Scientific, Victoria, Australia) and antibiotics, penicillin and streptomycin (GibcoTM, Life Technologies, Victoria, Australia; Catalog number—15140122), at 37 °C and 5% CO_2_. The cells were transduced with the modified BL2T vector from L2T containing tomato fluorescent protein and the luciferase 2 gene ([34]; the L2T vector was a kind gift from Dr. Michael F. Clarke, Stanford University, CA, USA), and annotated as BL2T-MDAMB468. Therapy-resistant MDA-MB-468 cells were generated in Adrian Wiegman’s research lab by culturing with doxorubicin (50 nM) and docetaxel (0.5 nM) over 6 months.

### 2.2. ‘Polarity Pool’ shRNA Library 

The ‘Polarity Pool’ shRNA library contains 4020 hairpins targeting 1677 genes related to epithelial cell polarity systems, breast cancer stem cells, epithelial mesenchymal plasticity, cell migration, and metastasis, and includes the entire breast cancer cell line ‘Basal B discriminator’ genes [35]. Lentiviral shRNAs were sourced from Open Biosystems (Dharmacon RNAi Technologies Decode Pooled Libraries, pGIPZ-mir30 backbone [36]) and transduced into MDA-MB-468 cells at the Victorian Centre for Functional Genomics (VCFG), Peter MacCallum Cancer Centre, Parkville, VIC, Australia. Lentivirus was produced using the pGIPZ polarity library DNA (1 μg/μL) together with lentivirus packaging and viral envelope plasmids, and transfected into HEK293T packaging cells using FuGENE reagent (Roche, Castle Hill, NSW, Australia). Forty-eight hours after transfection, media containing shRNA-encoding lentivirus particles were collected, aliquoted, and stored for further use [36,37].

### 2.3. ‘Polarity Pool’ shRNA Library Infection of BL2T-MDAMB468 Cells

BL2T-MDAMB468 cells were infected with the lentiviral shRNA pool at a multiplicity of infection of 0.320. After 2 days of selection in 2 mg/mL puromycin-containing medium to eliminate uninfected cells, puromycin-resistant BL2T-MDAMB468 cells were expanded for an additional 2 days. The transduced BL2T-MDAMB468 cells were then split into aliquots of 2 × 10^6^ cells each and frozen for future screens, including the screen described here.

### 2.4. Dose Determination for Growth Inhibition

Transduced BL2T-MDAMB468 cells were seeded in 96-well plates at 10,000 cells/well in 100 µL of growth medium. After overnight incubation, media was topped up to the appropriate concentrations with each of the three selected drugs. Drugs were 3-fold serially diluted in media across the range from 100 – 0.001µM. After 72 h of incubation, cell viability was measured with the resazurin-based Alamar Blue assay (#R7017, Sigma-Aldrich, St. Louis, MO, US) and the fluorescence intensity in each well was measured after 1 h using a top-reading fluorescent plate reader (FLUO Star Omega, BMG LABTECH, Mornington, VIC, Australia) with excitation at 544 nm and emission at 590 nm. The treatments were performed in triplicate (Figure S1A). For each drug, a concentration that corresponded to 10% growth inhibition (EC_10_) was used for subsequent ‘drop-out’ shRNA screens. 

### 2.5. RNA Extraction, cDNA Synthesis, and Reverse Transcriptase-Quantitative PCR (RT-qPCR) 

RNA was extracted using TRIzol (Life Technologies, VIC, Australia) and subsequent steps were performed as per the Bioline Isolate II RNA Micro kit manufacturer’s instructions (Bioline, Alexandria, NSW, Australia). cDNA was synthesized using the SensiFAST^TM^ cDNA Synthesis kit from Bioline. SYBR Green Master Mix was used to perform qRT-PCR in a ViiA7 Real-Time PCR system (Applied Biosystems, Carlsbad, CA, USA) and analysis was performed using QuantstudioTM Real-Time PCR software v1.1 (Applied Biosystems, Life Technologies, VIC, Australia). The primer sequences are listed in Table S1.

### 2.6. Western Blotting 

Total cell lysates of therapy-resistant MDA-MB-468 and matched sensitive cells were made using RIPA buffer (10mM Tris-HCl pH 7.6, 10mM NaCl, 3mM MgCl2, 1% Nonidet P-40, 1 X Protease Inhibitor tablet (Roche)). Protein levels were quantified using the Pierce^TM^ BCA Protein Assay Kit (Thermo Scientific, VIC, Australia). In total, 20 µg of total protein from each sample was prepared with sample-reducing buffer (2M urea, 2% SDS (sodium dodecyl sulfate), 0.125M Tris HCl, 0.1M DTT (dithiothreitol), and bromophenol blue) at a ratio of 3:1 (lysate: reducing buffer) and resolved on an SDS PAGE gel with Bolt™ MES SDS running buffer (Invitrogen, Waltham, MA, USA). The samples were subsequently transferred onto nitrocellulose membranes (BioTrac NT, Pall Life Sciences, New York, NY, USA) and blocked using 5% bovine serum albumin (BSA, Sigma, St. Louis, MO, USA). Blots were probed with mouse anti-TGFB2 mAb (clone 8607, R&D Systems), rabbit anti-TGFBR1 (Invitrogen, Waltham, MA, USA), goat anti-TGFBR2 (R&D Systems), and mouse anti-tubulin mAb (clone DM1A, Sigma, St. Louis, MO, USA). Membranes were then scanned on the Odyssey^®^ CLx imaging system (LI-COR Biosciences, Lincoln, NE, USA) using IRDye^®^ 680RD and IRDye^®^ 800CW secondary antibodies (LI-COR Biosciences, Lincoln, NE, USA).

### 2.7. Drug Treatment for Selection of ‘Polarity Pool’-Enriched Hairpin Library of BLT-MDAMB468 Cells

**Figure 1:**
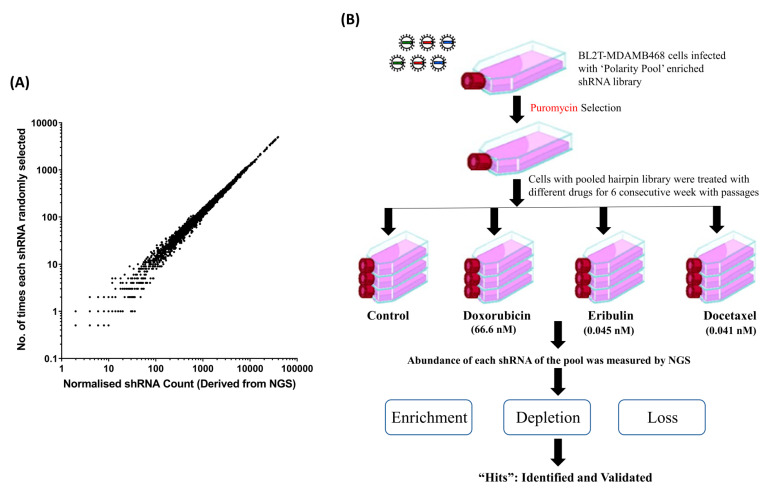
(**A**) Mathematical modelling to simulate the distribution of sampling error, in regard to previously obtained hairpin abundance in breast cancer cell line PMC42-LA induced with the same Polarity Pool-enriched library. (**B**) Workflow for the Polarity Pool-enriched short hairpin RNA (shRNA) library screen in breast cancer cell line MDA-MB-468 coupled with Ion Torrent sequencing to study genes involved in resistance against three chemotherapeutic drugs.

Three million cells from the first generation of cellular growth after thawing of the lenti-viral-infected library was saved as the control (“C0”) sample and the rest of the infected BL2T-MDAMB468 cells were seeded in 12 ^@^ T175 flasks at 2 × 10^6^ cells with 13 mL of growth media. A minimum number of 1 × 10^6^ cells account for the representation of at least one copy of each hairpin (Figure 1A). On the day after seeding, the three selected drugs (at EC_10_) were added to separate flasks in triplicate alongside three untreated flasks, which served as controls. Cells were incubated for a week and trypsinised, after which 2 × 10^6^ cells were again reseeded in a fresh flask and the remaining cells were frozen. The same treatments were again added the next day at the same EC_10_ concentration for each drug. This cycle was repeated for 6 consecutive weeks and 3 × 10^6^ cells were selected from the last sample for genomic DNA extraction. The rest were stored at −80 °C. The final time point was assessed for each screen. 

### 2.8. Genomic DNA Extraction and shRNA Amplification

Genomic DNA was extracted from each individual replicate sample using a Qiagen QIAamp DNA Blood Mini Kit (Qiagen, VIC, Australia; Catalog Number – 51106) according to the manufacturer’s instructions. Extracted DNA was quantified, barcoded, and PCR amplified. Adapter sequences and indexing primers used with the next-generation shRNA libraries are provided in Table S2. PCR amplification of shRNA was performed using an in-house SYBR Green PCR Master Mix. The 2× SYBR Master Mix (20 mL) comprised nuclease-free water (12.04 mL), DMSO (3.2 mL), 10× Gold PCR Buffer (Applied Biosystems, Waltham, MA, USA, 4 mL), 1M Mg(CH_3_COO)_2_ (100 µL), 100 mM dNTPs (80 µL of individual stocks), 6-ROX (20 ng/mL; 40 µL), 1/100 SYBR Green 1 (Molecular Probes, 100 µL), and AmpliTaq Gold (Applied Biosystems, Waltham, MA, USA, 200 µL). The cycling parameters used were 95°C for 5 min, 35 cycles of 95°C for 15 s, 60°C for 30 s and 72 °C for 1 min, and final extension at 72 °C for 10 min. The PCR product was verified on a 1.5% agarose gel to confirm a preponderance of a ~634-bp PCR product (53 nt for the forward primer sequence, 513 nt for the vector-including hairpin sequence, and 68 nt for the reverse primer sequence (including 30 nt for adaptor and 9 nt for barcode) (Figure 1B for PCR product). PCR products were purified using a Bioline PCR & Gel Purification Kit (Bioline, Eveleigh, NSW, Australia; cat: Bio-52029), quantified, pooled, and sent to Thermo Fisher Scientific, Melbourne, Victoria, Australia for Ion Torrent sequencing.

### 2.9. Data Analysis

Student’s t-tests were performed on normalised read counts to compare the triplicate drug-treated samples from the three selected drugs with the untreated control samples, to assess the enrichment, depletion, and loss of hairpins. Hairpins (synthetic lethal or rescuing the drug toxicity) altered by two-fold and with a significant *p*-value< 0.01 were considered as primary hits. Hairpins were also annotated for functional pathway enrichment using gene ontology and PANTHER [38] analysis of the hairpin libraries from untreated and doxorubicin-treated MDA-MB-468 cells. A gene expression connectivity mapping approach was also employed to identify perturbagens based on this data that may act synergistically with doxorubicin. The significant drop-down hairpins from doxorubicin-treated samples were compared to the reference drug expression profiles in the CMap v2.1 clue.io database (https://clue.io/) [39]. Pharmacologic classes of compounds with statistically significant negative enrichment scores in connection to the query gene signature were selected for further laboratory validation.

### 2.10. Drug Combination Experiments 

MDA-MB-468 cell viability was tested in response to the drug combinations in a matrix format, using combinations of doxorubicin with either SB525334 (TGF-β inhibitor), BGJ398 (FGFR inhibitor), or RITA (MDM2 inhibitor) with half-log fold serial dilutions. The same inhibitors were also tested in combination with docetaxel and eribulin. Two-fold serial dilutions of SB525334 were also tested with or without the presence of 1 µM P-glycoprotein inhibitor Tariquidar (Selleckchem, Redfern, NSW, Australia), 50 nM doxorubicin, and 0.5 nM docetaxel for comparing therapy-resistant MDA-MB-468 and matched sensitive cells. MDA-MB-468 cells (1 × 10^5^) were seeded overnight in growth media in a 96-well plate format and drug combinations were added after 24 h and incubated for a further 72 h. 

The cell viability was measured with the resazurin-based Alamar Blue assay as described above. The drug treatments were performed in triplicate. Drug combination synergy was assessed by utilizing the zero interaction potency model of SynergyFinder (https://synergyfinder.fimm.fi/), wherein the module compares the observed joint inhibition level at each dose combination to the expected combination effect [40,41].

## 3. Results

### 3.1. Mathematical Modelling to Simulate the Distribution of Sampling Error in Regard to Hairpin Abundance in PMC42-LA Cells

Using the PMC42-LA cells previously transduced with the same ‘Polarity Pool’ shRNA library, computational modelling was performed to determine the magnitude and distribution of the sampling error in regard to hairpin abundance, in order to assess whether the proposed hairpin-enrichment experiments would likely have sufficient power. The probability of being sampled was determined for each individual hairpin in the library from its abundance as determined by previous NGS. In this example, random sampling of one million hairpins was modelled for the ‘Polarity Pool’-enriched hairpin library-transduced PMC42-LA cells (Figure 1A). These data demonstrate how sampling error varies with the relative abundance. Based on this result, the in vitro hairpin enrichment experiments performed on samples of 1 × 10^6^ hairpin-containing cells were considered to be of sufficient size for hairpin enrichment to overcome sampling error. We therefore used 2 × 10^6^ cells for the corresponding drug screens of the ’Polarity Pool’ shRNA library in MDA-MB-468 cells.

### 3.2. Hairpin Representation in Functional Pathways

**Figure 2:**
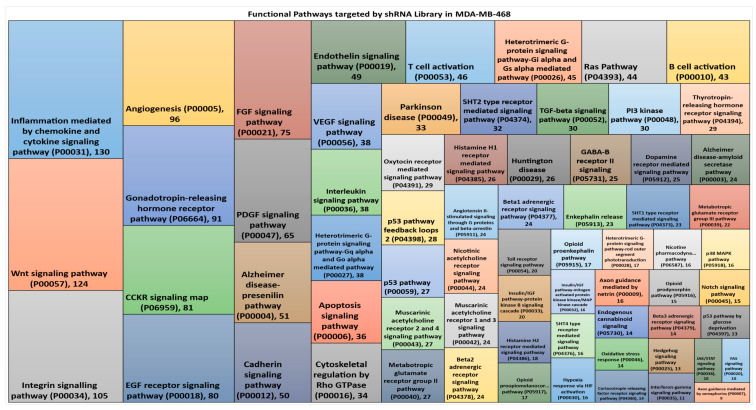
Gene ontology treemap representing the functional pathway enrichment from the genes targeted in the Polarity Pool-enriched hairpin library. The box size correlates to the total number of genes targeted by hairpins (also shown as the number within the box) belonging to the same functional pathway. Deduced hairpins targeting genes may well be represented in more than one pathway.

Hairpins for which the raw count value from the sequencing analysis was more than 100 in our untreated control samples were taken into consideration to screen for hairpin enrichment within the pathway analysis. The sequenced library deduced was found to comprise 2413 shRNAs targeting 1352 genes, instead of 4020 shRNAs specifically selected and transduced for 1677 genes. Key functional pathways affected by these hairpins were identified using gene ontology (GO) and PANTHER classification systems, and were found to represent enrichment for 68 signalling pathways. The number of gene targets identified can overlap between different signalling pathways. Treemap-computed visualisation shows that the largest groups of these hairpins target inflammation mediated by cytokine and chemokine signalling (130 gene targets), WNT signalling pathway (124 gene targets), integrin signalling pathway (105 gene targets), and EGF receptor signalling pathway (80 gene targets) (Figure 2). The custom-designed boutique library therefore contains an intrinsic bias that limits the identification of functional genes involved in EMP-associated chemoresistance, which would be the subject of further study.

### 3.3. ‘Polarity Pool’-Associated shRNA Hairpin Lethality Screen Performance

**Figure 3:**
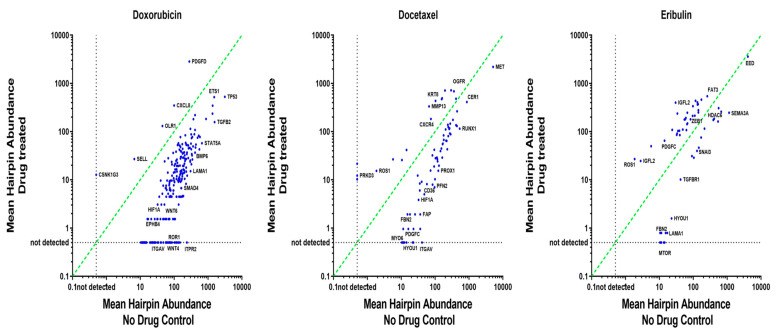
Distribution of hairpin abundance of the normalised reads of shRNAs in drug-treated versus untreated control from Polarity Pool-enriched hairpin library-induced BLT-MDAMB468 cells. Three scatterplots of the mean-normalised reads per shRNA for the shRNA found to be significantly different between the drug-treated versus untreated cells are shown. The bold diagonal line represents a ratio of 1 for the average amount of shRNA from each of the two conditions. Representative genes corresponding to the shRNA hits depleted during drug treatment are reflected on the right side of the diagonal, and those enriched during drug treatment are reflected on the left side of the diagonal. shRNAs lost during drug treatment are also represented on the horizontal dotted lines.

Using the established workflow (Figure 1B), the screen was conducted for hairpins that were depleted in cells showing drug resistance in MDA-MB-468 cells. This loss of hairpin representation in the screen allowed us to identify the candidate “rescuer” genes associated with resistance to chemotherapy, such that the knockdown of these genes should result in severe growth inhibition in the drug assay. The focus of this study was to identify those target genes that can overcome doxorubicin, docetaxel, and/or eribulin resistance in MDA-MB-468 cells. In total, 259 hairpins were identified to be dropped out or depleted and 5 hairpins were found to be enriched from the doxorubicin drug screen assay at *p*-value < 0.01. The assessment of the depleted hits as genes in the pathway enrichment assessed by GO/Panther is shown in Figure S2. However, none of the differences in the pathway enrichment assessed using Fischer’s exact test were statistically significant after adjusting for multiple testing. From the docetaxel drug screen assay, we identified the depletion of 66 hairpins and enrichment of 18 annotated hairpins at *p*-value < 0.01, and from the eribulin screen, 25 depleted hairpins and 32 enriched hairpins were identified at *p*-value < 0.01 (Figure 3). Relative fold enrichment and depletion of hairpins as log_2_ ratio and *p*-value < 0.01 are presented in Tables S3, S4, and S5, respectively, for all the three drug treatments versus untreated control cells. From all three independent drug screen assays, we identified the depletion of TGFB2, RUNX1, CCDC80, and HYOU1 hairpins to be in common.

Significant hits deduced from our hairpin library with doxorubicin treatment at *p*-value of <0.01 belong to mitotic spindle assembly (*EPB41, RASA1, APC,* and *ARHGEF7*); p-53 pathway (*TP53, HDAC1, CCNE1, APP, PRKAB1*), EMT (*VIM, INHBA, LAMA1, SFRP4, COL4A1*); FGF signalling pathway (*MAPK8, PPP2CB, FGFR3, FGF16, MAP2K6, MAP2K7, PIK3C2A, FGF5*); TGF-β signalling pathway (*MAPK8, SMAD4, INHBA, TGFB2, BMP6, BMP5*), and the genes mediating apoptosis (*TGFB2, LEF1, APP*). In the case of docetaxel treatment, the significant hits belong to genes encoding components of the integrin signalling pathway (*LAMA2, RAPGEF1, RND3, COL23A1, PTK2B, ITGAV*), Wnt signalling pathway (*GNG12, CDH10, MYH7, MYH8, HLTF, CER1, GNB1*), EMT (*ITGAV, FAP, PFN2*), and also for genes involved in cytoskeletal regulation by Rho GTPase (*PFN2, MYH8, DIAPH2*).

### 3.4. CMap Analysis Revealed Potential Inhibitors to Be Synergistic with Doxorubicin 

Evaluation of our ‘Polarity Pool’-enriched shRNA library data was performed in silico utilising Connectivity Map (CMap) to identify pharmaceutical perturbagen as relevant inhibitors that could enhance doxorubicin activity. This correlation-based pattern-matching software utilises the top 150 input gene signatures from our shRNA library analysis. Since the number of hairpins identified as depleted were less than 150 in case of docetaxel and eribulin, the shRNA drop-out screen library data were analysed further only for doxorubicin using the CMap. Based on the degree of difference between doxorubicin and the pharmaceutical perturbagen, a connectivity score was assigned, and the negative enrichment score was used to identify a perturbagen inducing a synergistic effect with doxorubicin. The top negative enrichment scores belong to classes of MDM inhibitors, TGF-β receptor inhibitors, and FGFR inhibitors (Table 1, Table S6). The score of 97.5 with doxorubicin (among the topoisomerase inhibitor set of compounds) serves as positive validation from the CMap analysis. 

### 3.5. SB525334 and RITA Inhibitors Synergistically Inhibited MDA-MB-468 Cell Viability in Combination with Doxorubicin

The efficacy of the selective inhibitors: SB525334 (TGFBR inhibitor), BGJ398 (FGFR inhibitor), and RITA (MDM inhibitor) identified using CMap were evaluated in dual combinations. The inhibitors were not only validated for doxorubicin but also for docetaxel or eribulin in MDA-MB-468 cell cultures. 

**Figure 4:**
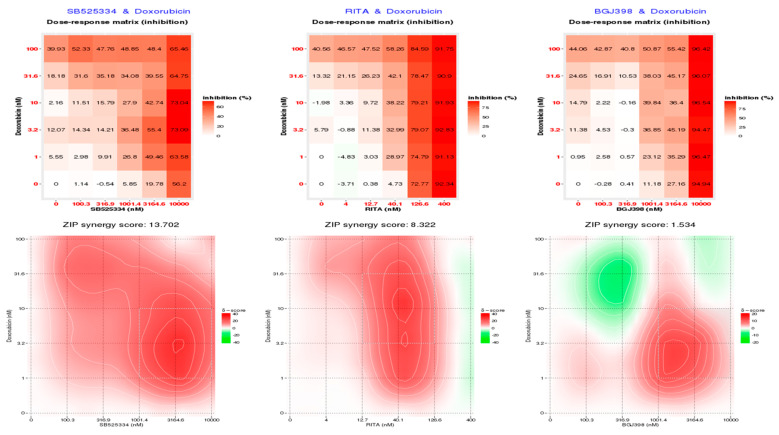
Dual combinations of doxorubicin with the inhibitors SB525334 and RITA synergistically inhibit MDA-MB-468. Cells were treated with an increased dose of doxorubicin up to EC50 together with either SB525334 (TGFBR inhibitor), RITA (MDM inhibitor), or BGJ398 (FGFR inhibitor). 2-D contour plots indicate areas of synergistic inhibition of cell viability by the red colour and antagonism by the green colour. Synergistic inhibition of MDA-MB-468 cell viability was found in the case of doxorubicin with SB525334 and RITA, while no synergy was detected in combination treatment of BGJ398 with doxorubicin. The top panel shows the data as a heatmap representing the raw dose–response matrix data for the percentage of cell inhibition for drug combinations.

The dual combination of SB525334 with doxorubicin evaluated using SynergyFinder revealed synergy across all the dose ranges, with an overall synergy score of 13.7, and highest synergistic area score of 19.38 (Figure 4). The combinations of RITA and doxorubicin also induced synergistic inhibition of MDA-MB-468 cell viability with a synergy score of 8.32 and highest synergistic area score of 14.28. The landscape of the drug interaction scoring region depicts that the RITA-driven synergy is triggered at a concentration of 40 nM. However, no synergy was detected in the combination of the FGFR inhibitor BGJ398 with doxorubicin. The raw dose–response pattern (Figure 4) visualised as a heat map also denotes the EC50 to be above 3 µM for the FGFR inhibitor BGJ398, which suggests that the observed response is due to the inhibition of various other kinase receptors, rather than acting specifically on FGFR(s). The drug combination results for each inhibitor with docetaxel and eribulin compounds are also provided in Figure S3 and Table S7, where only the combination of SB525334 with eribulin acts synergistically, and the horizontal landscape indicates that eribulin acts as a stable efficacy boost for the SB525334 inhibitor. 

### 3.6. MDA-MB-468-Resistant Cells Display Enhanced TGF-β Expression and Can Be Sensitized Using SB525334 

**Figure 5:**
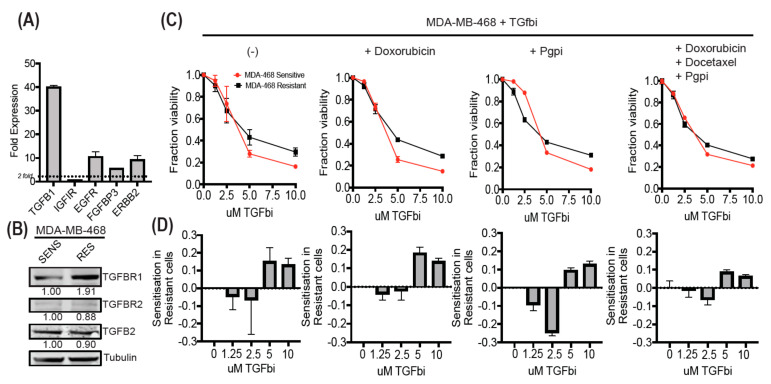
Low-dose targeting of TGF-β receptor 1 sensitizes chemoresistant MDA-MB-468. (**A**) Gene expression analysis of growth factor ligands and cell surface receptors in docetaxel/doxorubicin-adapted MDA-MB-468 (RES) cells as a ratio compared to matched sensitive MDA-MB-468 (SENS) cells. (**B**) Protein expression of TGF-β receptors (TGFBR1/2) and ligand (TGFB2) comparing matched MDA-MB-468-sensitive and -resistant cell lines. (**C**) TGF-β receptor 1 inhibitor SB525334 dose curves with the addition of doxorubicin, p-glycoprotein pump inhibitor (Pgpi), and the combination of doxorubicin (50 nM), docetaxel (0.5 nM), and Pgpi (1 µM). (**D**) The change in the sensitivity of the resistant cell line compared to sensitive cell line under the conditions in (**C**). All experiments were performed in triplicate and plotted as average +/- SEM.

Therapy-resistant MDA-MB-468 cells were obtained after adapting prolonged culture to the frontline combination of doxorubicin and docetaxel drug treatments. We compared the resistant cell line for gene and protein expression of appropriate growth factors receptors (Figure 5A,B; Supplementary Figure S4). Gene expression analysis of this line revealed enhanced TGFB1 expression by 40-fold, supporting our findings, and protein expression analysis of appropriate TGF-β receptors also reflected enhanced TGFBR1 expression in resistant cells. Dose curve analysis of a TGFBR1 inhibitor revealed the sensitisation of resistant cells at low doses but continued resistance at high doses (Figure 5C,D). This could be partially attributed to the known enhanced P-glycoprotein (Pgp) activity in the resistant line, which displayed increased sensitivity to TGFBR1 targeting in the presence of Pgp inhibitor at low doses. We suggest that the high dose response is due to the enhanced off-target cytotoxicity more readily observed in the sensitive cell line. The addition of doxorubicin or combination of doxorubicin and docetaxel did not enhance the targeting of TGFBR1, confirming our hairpin data.

## 4. Discussion

The drug activity on cancer cells is often modulated by several complex and not yet deciphered cellular mechanisms. The mechanism by which the EMP programmes are linked to drug resistance is not precisely understood; however, possible contributions include increased expression of drug efflux pumps, reduced cell division, and direct suppression of apoptotic/anoikis response pathways [3]. Various reports have confirmed the distinct molecular profile of EMT-derived cancer cells that are more resistant to chemotherapeutics [5,6,7,8,9,10,11,42]. Investigations of the association between the gene expression profiles of tumour samples and clinical responses of patients have generated a solid relationship between an EMT-associated gene expression signature and therapy resistance [3,4,43,44]. 

Efforts to characterise the mode of action of EMP-associated resistance through genetic perturbation, such as functional screening with genome-wide- or polarity pool-enriched shRNA libraries, may uncover downregulated or upregulated genes responsible for cell survival under drug stress. Our hairpin library is composed of various immunomodulators, cytokines, apical junction proteins, growth factors, and several downstream signalling gene products that mediate and/or have significant roles in EMP. The findings illustrated in Tables S2, S3, and S4 highlighted the genes relevant in the role of ‘rescuers’ under the drug stress from doxorubicin, docetaxel, and eribulin. This strategy also helps in the validation of the developed therapeutics against selected malignancy drivers, and it sets the stage for clinical testing of novel therapeutic biomarkers, before the initiation of preclinical validation and clinical trials. Such shRNA dropout viability screens to identify genes that sensitise cells to a drug’s lethal effect when inhibited have been conducted successfully in a number of studies against gemcitabine in a pancreatic cancer cell line [45], gefitinib in a non-small cell lung cancer [46], epirubicin in gastric cancer cells [47], anti-inflammatory bardoxolone methyl (CDDO-Me) drug in metastatic melanoma [48], and in ABT-737-treated lymphoma cells [49]. However, one of the significant drawbacks of this study owing to the EMP-focused custom-designed boutique shRNA library is that we were not able to analyse the drug resistance-related attenuated functional pathways. Since the number of hits deciphered in case of docetaxel and eribulin were lower, Figure S3 illustrated the pathway enrichment genes responsible for doxorubicin resistance (owing to 235 hits identified in the GO/Panther database). Further, due in part to the overall EMP bias, the identified pathways assessed from hairpin-negative enrichment analysis did not surpass the threshold *p*-value <0.05.

The identification of various common gene targets from the three different hairpin drop-out drug screenings, such as depletion of TGFB2, RUNX1, CCDC80, and HYOU1 hairpins, further warrants functional shRNA validation assays. Although the selected hairpins as target genes that confer drug sensitivity can be investigated using independent siRNAs or stably silenced shRNA studies, our results from the drug combinations revealed using Connectivity Map and tested using SynergyFinder reflect the potential to accurately predict a pattern that is related to their underlying target interactions. CMap is a genomic screening tool for linking genes associated with a selected phenotype with potential therapeutic agents [38,50]. Our results indicated that the MDM inhibitors (e.g., RITA) and inhibitors of TGFB or TGFBR (e.g., SB525334) used with doxorubicin showed synergistic interactions in drug combination assays, and that the administration of MDM inhibitors or TGF-β/TGF-βR inhibitors alongside doxorubicin may have the potential to improve the survival rate of breast cancer patients. The MDM inhibitors (which block p53–MDM interactions) alongside doxorubicin appear to be a promising strategy in curtailing drug resistance as previous studies have confirmed that the MDM inhibitor RITA enhanced chemosensitivity to doxorubicin [51,52]. Chiu and his group further observed that RITA mediates Stat3 and Erk suppression, which can be activated in apoptosis caused by doxorubicin [53]. 

Similarly, TGF-β/TGF-β receptor inhibitors are also considered as potential targets for breast cancer therapy alongside doxorubicin, as additional studies have confirmed that TGF-β blockade improves the distribution and efficacy of doxorubicin [54,55]. Additionally, inhibitors of TGF-β and its receptors are also being extensively evaluated alongside other chemotherapeutics. For example, LY2157299 is being actively trialled along with chemotherapeutics in recurrent malignant glioblastoma, metastatic pancreatic cancer, hepatocellular carcinoma, castration-resistant prostate cancer, and triple-negative breast cancer, reflecting the consideration that this inhibitor can assist in the treatment of various solid malignancies. Our data demonstrate that the gene expression involved in TGF-β signalling as an EMP inducer [56] might be mediating chemoresistance to doxorubicin treatment, such that using TGF-β inhibitor signalling alongside doxorubicin can potentially improve the effectiveness of this therapy. Interestingly, an extensive study of growth factor-associated gene expression changes in doxorubicin and docetaxel-resistant human breast cancer MDA-MB-468 cells showed a strong induction (~40-fold increase) of TGFB1 expression along with higher transcript expression of EGFR, FGFBP3, and higher TGFBR1 expression at the protein level. Further, drug assays highlight that these resistant cells can be sensitized to SB525334 in the presence of p-glycoprotein inhibition. 

The FGFR inhibitor BGJ398 was selected as an alternative to PD-173074 from our CMap studiesbecause of its design depending on PD-173074 and owing to its evaluation in phase III clinical trials. The failure of BGJ398 as a potent FGFR inhibitor to show effects alone or in drug combination experiments with doxorubicin in MDA-MB-468 cells could reflect a possible off-target effect of PD-173074. Ideally, we should repeat our analysis with the drug PD-173704 too, in case to check if it reflects the same off-target effect.

Although the shRNA studies have the potential to identify genes and pathways regulating the phenotype of interest (response to chemotherapy), successful gene-targeted therapy approaches for silencing genes for cancer treatment still meet many challenges [57]. The ability to evaluate the effective drug combinations using a multitude of experiments in complex and context-dependent cancer is a very daunting task. The mechanistic understanding of the drug suppression when used alone, as well as in relationships with other drugs, is another systematic challenge, and a third challenge is the statistical assessment of the synergies as different models can be implemented in combination experiments [40,58,59]. Fewer studies have been conducted at a large scale to determine the potential synergistic drug combinations [60,61,62]. Thus, a compendia of publicly available drug combination data is of the utmost need for its clinical utility but also the potential to strategically design these new combinations can be achieved using hairpin-based drop-out viability screening. Thus, understanding the scope of a drug’s activity at the cellular level using hairpin-based screening and the CMap approach can impact the validation of the potential drug target, and will guide in the development of improved therapies. These proven synergistic drug combinations may also allow lower dosing, and thus potentially limit the toxicity of the approved drugs. 

## Figures and Tables

**Table 1:** Significant perturbagens class obtained from Connectivity Map. Pharmacologic class of perturbagens reflected with enrichment scores above 90 (similar) and below -90 (opposing) deciphered via Connectivity Map from the hairpin screening for the drug doxorubicin.

CMap Classes	Sets of Compound Perturbagens with Enrichment Scores above 90 (Similar) and below -90 (Opposing)	Pharmacologic included Drug Numbers
Topoisomerase inhibitor	94.01	16
ATPase inhibitor	92.45	16
TGF beta receptor inhibitor	−92.12	4
FGFR inhibitor	−94.27	4
Bile acid	−94.89	4
MDM inhibitor	−99.78	4

## Supplementary Materials

The following are available online at https://www.mdpi.com/2072-6694/12/5/1123/s1, Figure S1: (A) Growth inhibitory effects of doxorubicin, docetaxel, eribulin and inhibitors SB525334, RITA and BGJ398 in MDA-MB-468 cells after 72 hours exposure. (B) Agarose gel electrophoresis for the product verification of the amplified PCR product of 634 bp to be sent for hairpin sequencing from the all the thirteen samples, Figure S2: Gene Ontology treemap representing the hits deduced from doxorubicin drug hairpin drop-out screen assay corresponding to their functional pathway enrichment. The box size correlates to the total number of genes significantly depleted in the assay (also shown as number within the box) belonging to the same functional pathway. Deduced hairpins targeting genes may well be represented in more than one pathway, Figure S3: (A) Heatmap representing the raw dose-response matrix data for the percentage of cell inhibition for the indicated drug combinations. (B) 2D contour plot from drug combination assays indicating areas of synergistic inhibition of cell viability by red colour and antagonism by green colour as depicted using SynergyFinder, Figure S4: Western blot analysis of whole protein lysates extracted from docetaxel/doxorubicin-adapted MDA-MB-468 (RES) cells and matched sensitive MDA-MB-468 (SENS) cells and analysed for the protein expression of TGF-β receptors (TGFBR1/2), ligand (TGFB2) and α-tubulin antibody. Table S1 - List of Primers used in RT-qPCR annotated with their Gene Symbols and forward and reverse primer sequence information. Table S2 - List of Primers used in qPCR for amplification and tagging barcodes for next generation sequencing. Table S3: List of annotated shRNA primary screen hairpin hits identified in doxorubicin-treated MDA-MB-468 cells. Table S4: List of annotated shRNA primary screen hairpin hits identified in docetaxel-treated MDA-MB-468 cells. Table S5: List of annotated shRNA primary screen hairpin hits identified in docetaxel-treated MDA-MB-468 cells. Table S6: Set of compound perturbagens identified from CMap utilizing hits identified from doxorubicin. Table S7: Visualization of synergy scores for Drug Combinations.

## Abbreviations

BC	Breast cancer
EMP	Epithelial mesenchymal plasticity
CMap	Connectivity Map
shRNAs	short hairpin RNAs
EMT	Epithelial mesenchymal transition
CSC	cancer stem cells
RNAi	RNA interference
siRNA	small interfering RNA
NGS	Next generation sequencing
TGFBR	Transforming growth factor β receptor
FGFR	Fibroblast growth factor receptor
EGF	Epidermal growth factor
MDM	Murine double minute
WNT	Wingless-related integration site
