# Supplementary material for: Identifying Therapies to Combat Epithelial Mesenchymal Plasticity-Associated Chemoresistance to Conventional Breast Cancer Therapies Using An shRNA Library Screen
Source: Cancers (Basel). 2020 Apr 30;12(5):1123. doi: 10.3390/cancers12051123 (PMC7281530; doi:10.3390/cancers12051123)
Supplement: Supplementary file 1 [file cancers-12-01123-s001.pdf]

Supplementary Figure 1

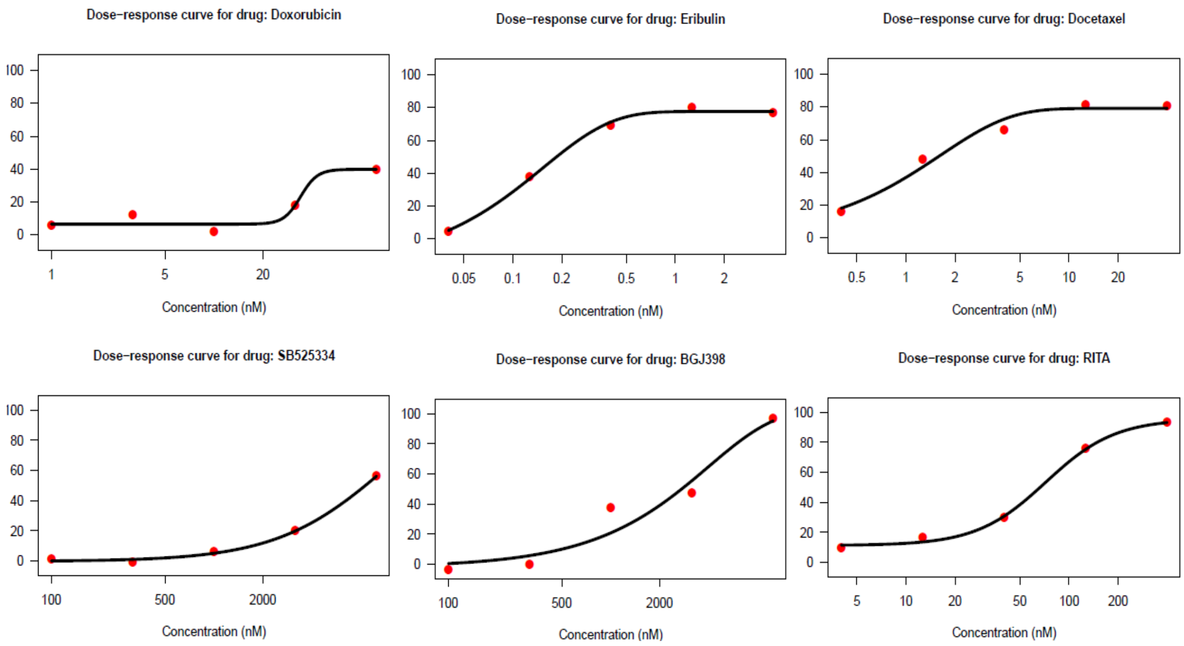

(A) Dose-response curves for drugs: doxorubicin, docetaxel, eribulin and inhibitors: SB525334, BGJ398, RITA. *Y scale is percentage of cell death*

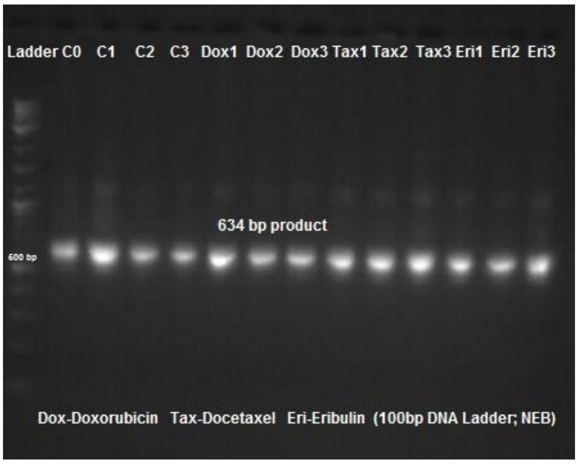

(B) Agarose gel electrophoresis for the product verification of the amplified PCR product of 634 bp to be sent for hairpin sequencing from the all the thirteen samples.

Supplementary Figure 2

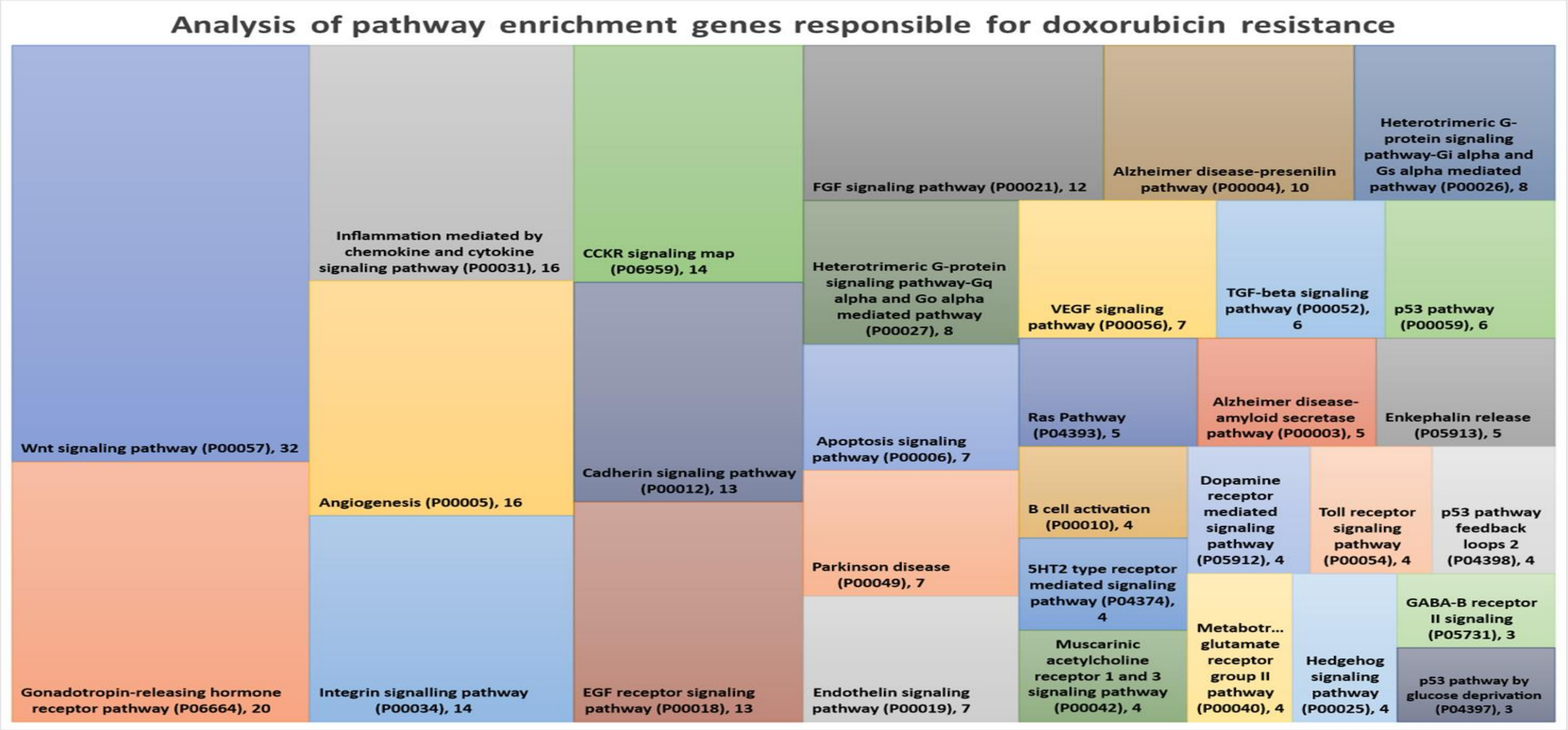

Supplementary Figure 2: Gene Ontology treemap representing the hits deduced from doxorubicin drug hairpin screen assay corresponding to their functional pathway enrichment. The box size correlates to the total number of genes significantly depleted in the assay (also shown as number within the box) belonging to the same functional pathway. Deduced hairpins targeting genes may well be represented in more than one pathway.

Supplementary Figure 3

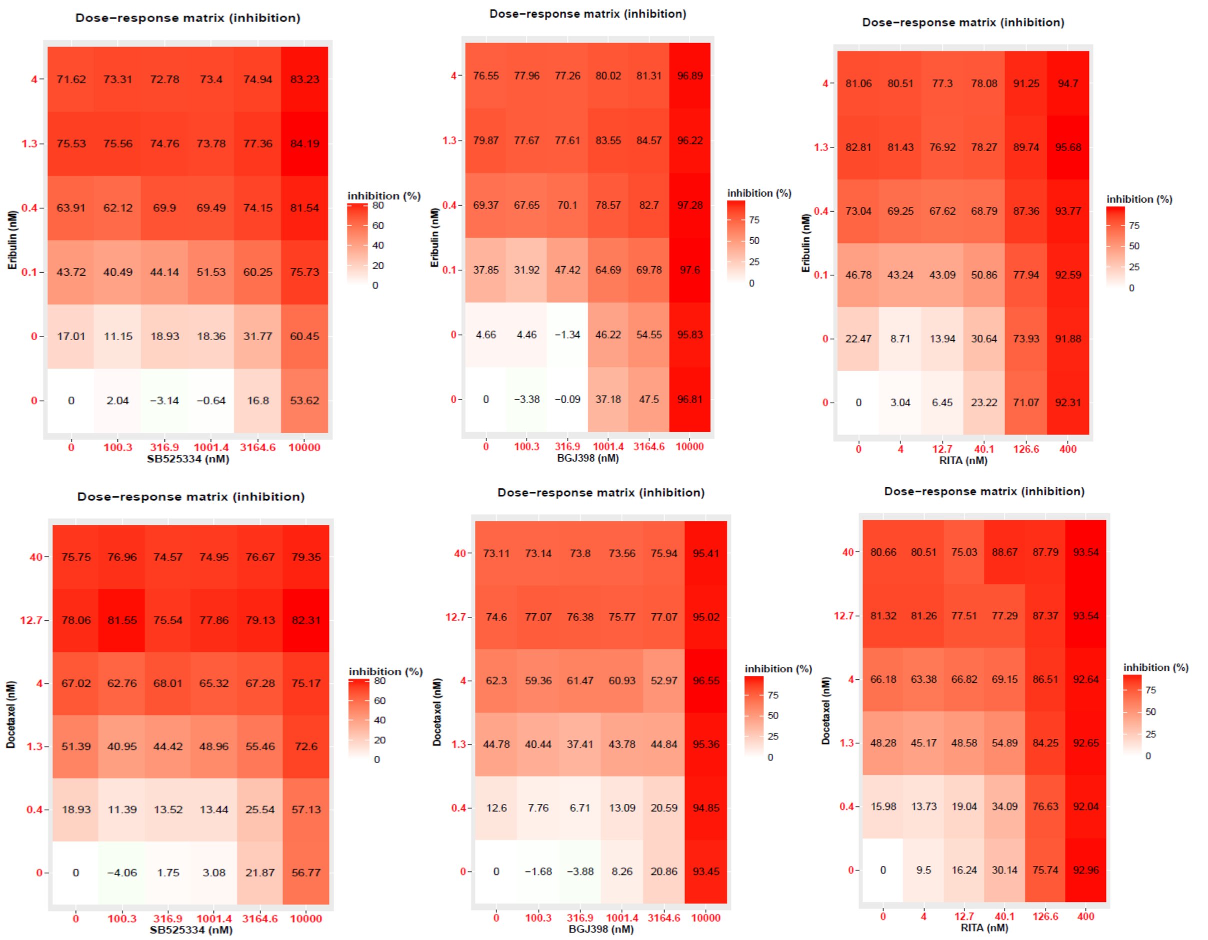

(A) Heatmaps representing the raw dose-response matrix data for the percentage of cell inhibition for the indicated drug combinations.

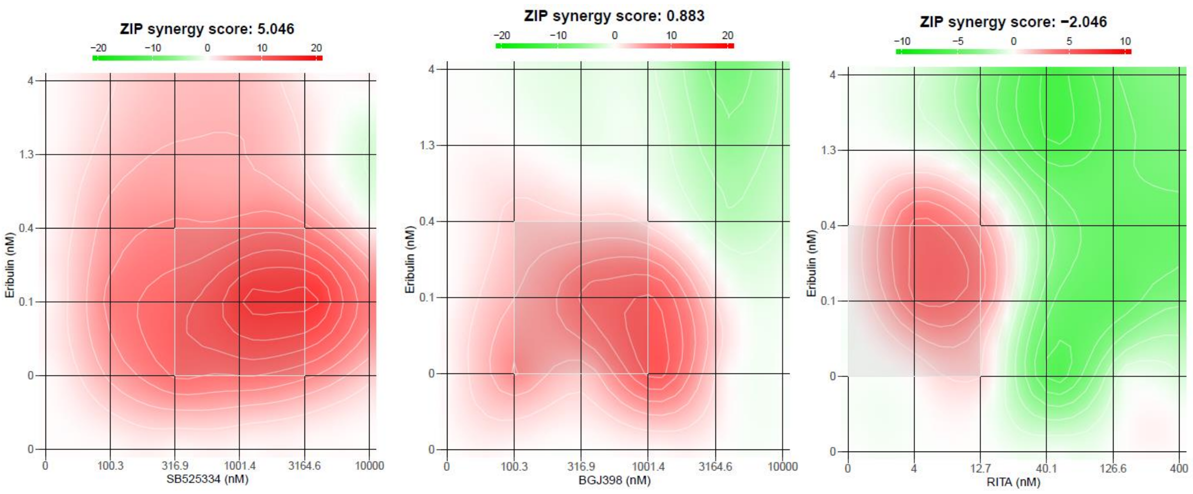

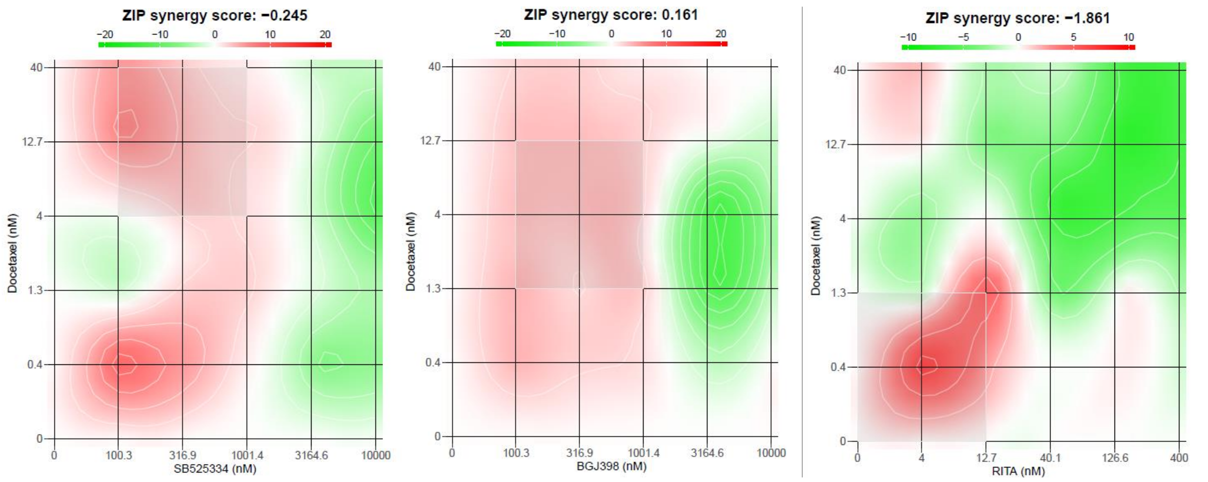

(B) 2D contour plots from drug combination assays indicating areas of synergistic inhibition of cell viability by red colour, and antagonism by green colour, as depicted using SynergyFinder.

Supplementary Figure 4

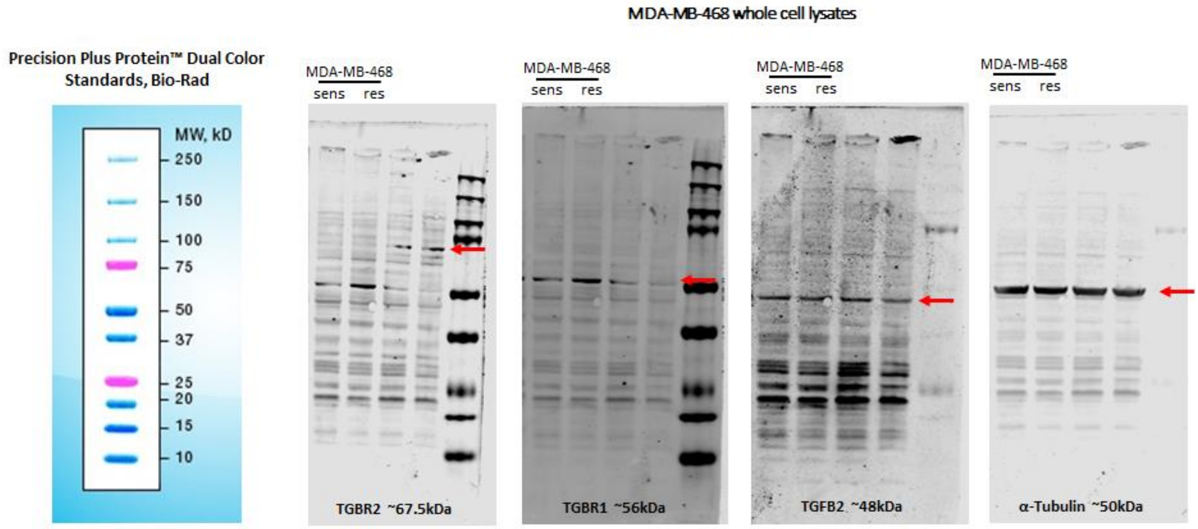

Western blot analysis of whole protein lysates extracted from docetaxel/doxorubicin-adapted MDA-MB-468 (RES) cells and matched sensitive MDA-MB-468 (SENS) cells and analysed for the protein expression of TGF-β receptors (TGFBR1/2), ligand (TGFB2) and α-tubulin antibody.

Supplementary Table S1 - List of Primers used in RT-qPCR annotated with their Gene Symbols and forward and reverse primer sequences information

Gene Symbol	Forward Primer Sequence	Reverse Primer Sequence
TGFB1	CGCGGGACTATCCACCTGCAA	CGGTCGCGGGTGCTGTTGTA
EGFR	GGACTGAAGGAGCTGCCCATGAGAAAT	ATAACCAGCCACCTCCTGGATGGTC
FGFBP3	CAGGGAGCCGGCTAAAACAGACTAC	AAAGAGCATTCCCCAGGACCGAGTT
IGF1R	CGACTGACCTGTCTTTGGAACCAGAACAT	CCTGGTCCCCCAGCAAGAAGCAAA
ERBB2	CTATGCCCTGGCCGTGCTAGACAAT	GCTGGATCAAGACCCCTCCTTTCAAGAT
L32	CAGGGTTCGTAGAAGATTCAAGGG	CTTGGAGGAAACATTGTGAGCGATC

Supplementary Table S2 - List of Primers used in qPCR for amplification and tagging barcodes for next generation sequencing

IonX_trP1_pGIPz	CCT CTC TAT GGG CAG TCG GTG ATC GTA TCC ACA TAG CGT AAA AGG AGC AAC AT
IonX_01_pGIPz	CCA TCT CAT CCC TGC GTG TCT CCG ACT CAG CTA AGG TAA CGA TGC AAG CCC GGT GCC TGA GTT TGT TT
IonX_02_pGIPz	CCA TCT CAT CCC TGC GTG TCT CCG ACT CAG TAA GGA GAA CGA TGC AAG CCC GGT GCC TGA GTT TGT TT
IonX_03_pGIPz	CCA TCT CAT CCC TGC GTG TCT CCG ACT CAG AAG AGG ATT CGA TGC AAG CCC GGT GCC TGA GTT TGT TT
IonX_04_pGIPz	CCA TCT CAT CCC TGC GTG TCT CCG ACT CAG TAC CAA GAT CGA TGC AAG CCC GGT GCC TGA GTT TGT TT
IonX_05_pGIPz	CCA TCT CAT CCC TGC GTG TCT CCG ACT CAG CAG AAG GAA CGA TGC AAG CCC GGT GCC TGA GTT TGT TT
IonX_06_pGIPz	CCA TCT CAT CCC TGC GTG TCT CCG ACT CAG CTG CAA GTT CGA TGC AAG CCC GGT GCC TGA GTT TGT TT
IonX_07_pGIPz	CCA TCT CAT CCC TGC GTG TCT CCG ACT CAG TTC GTG ATT CGA TGC AAG CCC GGT GCC TGA GTT TGT TT
IonX_08_pGIPz	CCA TCT CAT CCC TGC GTG TCT CCG ACT CAG TTC CGA TAA CGA TGC AAG CCC GGT GCC TGA GTT TGT TT
IonX_09_pGIPz	CCA TCT CAT CCC TGC GTG TCT CCG ACT CAG TGA GCG GAA CGA TGC AAG CCC GGT GCC TGA GTT TGT TT
IonX_10_pGIPz	CCA TCT CAT CCC TGC GTG TCT CCG ACT CAG CTG ACC GAA CGA TGC AAG CCC GGT GCC TGA GTT TGT TT
IonX_11_pGIPz	CCA TCT CAT CCC TGC GTG TCT CCG ACT CAG TCC TCG AAT CGA TGC AAG CCC GGT GCC TGA GTT TGT TT
IonX_12_pGIPz	CCA TCT CAT CCC TGC GTG TCT CCG ACT CAG TAG GTG GTT CGA TGC AAG CCC GGT GCC TGA GTT TGT TT
IonX_13_pGIPz	CCA TCT CAT CCC TGC GTG TCT CCG ACT CAG TCT AAC GGA CGA TGC AAG CCC GGT GCC TGA GTT TGT TT

Supplementary Table S3: List of annotated shRNA primary screen hairpin hits identified in doxorubicin-treated MDA-MB-468 cells

Screening Hit	Log_2 (doxorubicin/control)	p-value
ITPR2	-8.890	0.001826
ZFPM2	-8.204	0.00099
FAT4	-8.068	0.00090
TUBD1	-7.930	0.00258
BIRC5	-7.843	0.00391
COL12A1	-7.839	0.00599
ROR1	-7.700	0.00313

GLT8D2	-7.682	0.00039
PSMB2	-7.432	0.00688
H2AFY	-7.404	0.00487
NTRK1	-7.290	0.00211
MAP2K5	-7.245	0.00678
MMP10	-7.218	0.00001
APP	-7.160	0.00000
TEK	-7.154	0.00252
VIM	-7.109	0.00776
WNT4	-6.973	0.00032
MYL2	-6.933	0.00057
AC097532.1	-6.915	0.00392
TMSB4XP4	-6.844	0.00658
BBS4	-6.817	0.00208
BMP5	-6.580	0.00276
ITGAV	-6.361	0.00420
BBS4	-6.355	0.00015
KLF10	-6.328	0.00459
CCDC80	-6.322	0.00008
CCL2	-6.116	0.00957
PARVA	-6.101	0.00557
DAB2	-6.099	0.00200
RP11-140L24.4	-6.034	0.00994
STXBP5L	-6.018	0.00353
CCL7	-5.903	0.00154
GCFC2	-5.836	0.00002
RHOH	-5.739	0.00001
MERTK	-5.688	0.00015
PSMD6	-5.680	0.00324
TIAM1	-5.575	0.00233
COL4A1	-5.573	0.00838
FYB1	-5.516	0.00606
HYOU1	-5.516	0.00606
SYNPO2	-5.504	0.00779
TRHDE	-5.464	0.00005
WNT9A	-5.463	0.00206
CCNE1	-5.454	0.00294
RIC8B	-5.454	0.00294
ROR2	-5.342	0.00003
RASGRP2	-5.328	0.00459
PPP2R4	-5.274	0.00002
STK25	-5.263	0.00216
MAPK8	-5.259	0.00658
SLC35D3	-5.246	0.00056
MAP2K7	-5.238	0.00048

FAT1	-5.167	0.00259
EPB41	-5.102	0.00025
MAP2K6	-5.009	0.00151
MMP20	-4.977	0.00681
COL21A1	-4.959	0.00397
FYB1	-4.934	0.00019
PLOD2	-4.934	0.00019
ANAPC7	-4.934	0.00019
FRAS1	-4.929	0.00129
ARHGEF11	-4.843	0.00150
ITGAV	-4.843	0.00150
TGFB2	-4.843	0.00150
PTK2B	-4.843	0.00150
FCGR2A	-4.767	0.00306
MS4A4A	-4.747	0.00761
TAF5L	-4.732	0.00484
INSR	-4.732	0.00484
CELSR3	-4.732	0.00484
LIN7A	-4.730	0.00937
FBXW7	-4.706	0.00263
PPID	-4.674	0.00113
F11R	-4.627	0.00226
CCL23	-4.627	0.00226
MPP6	-4.627	0.00226
NFKB1	-4.627	0.00226
DPP6	-4.627	0.00226
SMAD4	-4.609	0.00017
SCRG1	-4.585	0.00561
GNAI3	-4.514	0.00200
MAPK8	-4.514	0.00200
PRKD1	-4.514	0.00200
OR10AG1	-4.514	0.00200
MAP3K10	-4.514	0.00200
CSGALNACT1	-4.514	0.00200
PTK2B	-4.514	0.00200
MYL12A	-4.514	0.00200
LTBP2	-4.514	0.00200
TLN2	-4.514	0.00200
CDH10	-4.514	0.00200
FZD4	-4.514	0.00200
RUNX1	-4.514	0.00200
SP1	-4.400	0.00489
EPHB4	-4.391	0.00532
HLTF	-4.391	0.00532
ITPR2	-4.391	0.00532

MS4A6A	-4.391	0.00532
CACNA2D3	-4.391	0.00532
RCAN2	-4.391	0.00532
GAST	-4.391	0.00532
PRKCZ	-4.357	0.00720
LAMA1	-4.334	0.00421
GRHL1	-4.321	0.00171
CREB1	-4.285	0.00405
NGFR	-4.249	0.00063
ERBIN	-4.184	0.00160
BTRC	-4.121	0.00016
FGF16	-4.118	0.00220
BEND6	-4.092	0.00603
TRIM2	-4.090	0.00964
SPOCK3	-4.081	0.00939
WNT6	-4.070	0.00148
CLIC4	-4.018	0.00097
SLC9A1	-4.018	0.00031
DLG2	-3.996	0.00342
GAS2	-3.978	0.00023
DKK3	-3.946	0.00582
ERBIN	-3.944	0.00038
CREG1	-3.881	0.00971
MAP3K8	-3.876	0.00183
SEMA4B	-3.868	0.00788
KCTD15	-3.828	0.00910
CCL22	-3.825	0.00172
EPOR	-3.808	0.00031
ARL6	-3.807	0.00124
EPHB4	-3.775	0.00734
DCN	-3.770	0.00753
APC	-3.755	0.00023
CLIC5	-3.740	0.00594
ITGB6	-3.715	0.00653
GRK7	-3.714	0.00855
GNAS	-3.706	0.00435
HCRTR1	-3.701	0.00188
FZD5	-3.698	0.00798
APC	-3.687	0.00884
PPP2CB	-3.680	0.00841
STXBP5L	-3.670	0.00344
CCR1	-3.663	0.00764
FRAS1	-3.663	0.00698
PTCH1	-3.660	0.00955
CDH10	-3.655	0.00922

MAPK8	-3.647	0.00538
GREM1	-3.581	0.00058
UNC13A	-3.573	0.00473
MYO1A	-3.548	0.00030
STAT5A	-3.534	0.00566
GZMK	-3.525	0.00323
MAST3	-3.520	0.00415
ARHGEF3	-3.514	0.00218
BMP6	-3.503	0.00046
CDH18	-3.501	0.00814
LAMA1	-3.481	0.00198
AURKB	-3.478	0.00127
MYH13	-3.468	0.00262
HIF1A	-3.401	0.00130
PDGFC	-3.401	0.00130
RP1-179P9.3	-3.394	0.00594
TUBB2A	-3.389	0.00310
ARHGEF7	-3.365	0.00006
CLEC2B	-3.349	0.00430
PTGS2	-3.330	0.00521
TGFB2	-3.315	0.00001
COL6A3	-3.302	0.00172
TNFAIP6	-3.293	0.00434
ELL2	-3.276	0.00804
GH1	-3.226	0.00723
SFRP4	-3.221	0.00162
PRKCG	-3.214	0.00824
CDH19	-3.206	0.00809
RP11-439C15.6	-3.191	0.00458
CACNA1E	-3.172	0.00885
PIK3C2A	-3.146	0.00593
LRRC7	-3.136	0.00348
GRHL1	-3.118	0.00176
TEAD1	-3.094	0.00064
CDK13	-3.092	0.00747
HELLS	-3.075	0.00346
MS4A6A	-3.053	0.00860
MYH14	-3.039	0.00223
KCTD7	-3.032	0.00322
MOXD1	-3.031	0.00757
MAP3K10	-3.023	0.00507
MPP6	-3.000	0.00982
ELL2	-2.971	0.00971
DOCK11	-2.943	0.00533
PSMA4	-2.914	0.00939

INHBA	-2.910	0.00563
CD69	-2.908	0.00165
MPP7	-2.897	0.00155
PMP22	-2.876	0.00959
MYL1	-2.871	0.00917
PPP3R1	-2.845	0.00913
RP11-144L1.8	-2.833	0.00307
ADRBK2	-2.786	0.00073
SVEP1	-2.785	0.00291
NF2	-2.764	0.00804
UNC13D	-2.724	0.00615
GPSM2	-2.709	0.00539
AXIN2	-2.709	0.00310
SMC3	-2.698	0.00979
RUNX1	-2.697	0.00781
INSC	-2.685	0.00749
COL8A2	-2.684	0.00349
CREG1	-2.681	0.00782
PSMC5	-2.659	0.00221
PYGO2	-2.658	0.00767
KCNK13	-2.625	0.00628
TP53	-2.569	0.00416
ITGAX	-2.563	0.00430
RP5-877J2.1	-2.520	0.00883
PLN	-2.484	0.00668
PLAUR	-2.481	0.00971
PROX1	-2.464	0.00374
EPOR	-2.435	0.00260
GNG11	-2.421	0.00149
LEF1	-2.401	0.00757
RAPGEF4	-2.390	0.00259
PLS3	-2.369	0.00805
HDAC1	-2.342	0.00476
SERPINA1	-2.341	0.00117
PLK4	-2.336	0.00533
NR4A3	-2.300	0.00529
RASA1	-2.263	0.00082
RP11-107P7.6	-2.206	0.00652
RAB25	-2.202	0.00649
KLF10	-2.201	0.00963
STRADA	-2.162	0.00101
ANLN	-2.162	0.00858
NRG1	-2.158	0.00476
RP11-215I23.3	-2.113	0.00828
CACNA1S	-2.091	0.00459

VEGFA	-2.061	0.00656
XDH	-2.049	0.00558
KAT2B	-2.026	0.00667
BICC1	-1.996	0.00083
DDR2	-1.991	0.00080
FREM1	-1.980	0.00348
HDAC6	-1.942	0.00573
COL17A1	-1.905	0.00616
KIF20A	-1.863	0.00759
GLI1	-1.853	0.00700
PTK2B	-1.771	0.00119
PPP1R3C	-1.687	0.00281
PRKAB1	-1.663	0.00003
CREM	-1.570	0.00144
ETS1	-1.557	0.00045
YWHAB	-1.514	0.00265
FGFR3	-1.409	0.00631
POU5F1	-1.376	0.00653
GPR34	-1.346	0.00612
SFRP4	-1.277	0.00505
PMP22	-1.268	0.00249
RAP2B	-1.257	0.00851
FGF5	-1.225	0.00759
TTC8	-1.130	0.00937
PRKACB	-1.027	0.00293
CDH19	-0.993	0.00480
PPP3CC	-0.912	0.00706
OLR1	1.536	0.00085
CXCL8	1.798	0.00613
SELL	2.016	0.00778
PDGFD	3.341	0.00055
CSNK1G3	4.666	0.00232

Supplementary Table S4: List of annotated shRNA primary screen hairpin hits identified in docetaxel-treated MDA-MB-468 cells

Screening Hit	Log_2 (docetaxel/control)	p-value
ITGAV	-6.361	0.00420
RIC8B	-5.454	0.00294
GNB1	-5.245	0.00755
ITGAV	-4.843	0.00150

DPP6	-4.627	0.00226
GNG12	-4.582	0.00909
MADCAM1	-4.514	0.00200
PTK2B	-4.514	0.00200
CDH10	-4.514	0.00200
RUNX1	-4.514	0.00200
COL24A1	-4.391	0.00532
GAST	-4.391	0.00532
MYO6	-4.391	0.00532
FAP	-4.245	0.00913
HYOU1	-4.216	0.00789
PDGFC	-4.079	0.00038
PFN2	-3.793	0.00003
PSMD6	-3.739	0.00619
SCRIB	-3.573	0.00490
RND3	-3.411	0.00275
ZBTB33	-3.278	0.00057
CD36	-3.249	0.00458
EXOC4	-3.235	0.00590
HIF1A	-3.079	0.00270
PROX1	-3.012	0.00251
FBN2	-2.993	0.00398
TGFB2	-2.843	0.00150
PTPRJ	-2.819	0.00050
CBY1	-2.728	0.00066
RP11-446E9.1	-2.560	0.00507
ZC3HAV1	-2.502	0.00485
RP11-215I23.3	-2.410	0.00141
ERBIN	-2.316	0.00468
CLCN6	-2.301	0.00240
TIAM1	-2.238	0.00915
RUNX1	-2.211	0.00580
CCDC80	-2.143	0.00388
EPHB4	-2.130	0.00610
DIAPH2	-2.080	0.00502
SERPINF1	-1.972	0.00570
GLT8D2	-1.879	0.00571
RAPGEF1	-1.870	0.00549
WIF1	-1.789	0.00891
PRKAR2A	-1.773	0.00391
DDAH1	-1.759	0.00011
NUMB	-1.715	0.00232
COL23A1	-1.708	0.00949
BMP6	-1.611	0.00105
MYH7	-1.607	0.00991

IQCH	-1.603	0.00188
SLC9A1	-1.596	0.00952
KDM4C	-1.576	0.00845
HLTF	-1.418	0.00658
EIF2B4	-1.378	0.00886
FGF9	-1.329	0.00262
MET	-1.234	0.00164
RP11-64K7.1	-1.195	0.00905
SERPINA1	-1.192	0.00766
GAS2	-1.178	0.00116
ARFIP2	-1.101	0.00079
CER1	-1.078	0.00623
NUMA1	-0.983	0.00508
DDR1	-0.946	0.00458
CXCR6	-0.932	0.00517
MYH8	-0.882	0.00094
ZNF782	-0.781	0.00973
LAMA2	-0.762	0.00690
PPP3CC	0.211	0.00981
OGFR	0.926	0.00723
STAC	1.279	0.00900
PIEZO2	1.283	0.00325
CXCR4	1.285	0.00921
PDPK1	1.533	0.00281
KRT8	1.589	0.00562
PARD6B	1.610	0.00404
DIRAS3	1.862	0.00975
RNF168	2.078	0.00032
RCAN3	2.210	0.00219
MMP13	2.329	0.00578
ROS1	3.037	0.00636
LIMK2	4.358	0.00131
FYN	4.358	0.00131
ATF5	4.358	0.00131
PRKD3	4.605	0.00122
TP53	5.424	0.00050

Supplementary Table S5: List of annotated shRNA primary screen hairpin hits identified in docetaxel-treated MDA-MB-468 cells

Screening Hit	Log_2 (Eribulin/control)	p-value
MTOR	-4.843	0.00150
MMP11	-4.747	0.00761

FZD4	-4.514	0.00200
LAMA1	-4.437	0.00069
PSENEN	-4.391	0.00532
ITGA1	-4.391	0.00532
COL21A1	-4.391	0.00532
FBN2	-4.271	0.00050
HYOU1	-3.853	0.00977
RUNX1	-3.851	0.00393
MS4A6A	-3.728	0.00932
SEMA3A	-2.215	0.00061
TGFBR1	-2.058	0.00390
TGFB2	-1.883	0.00150
SCN8A	-1.864	0.00079
GDF10	-1.737	0.00766
SNAI3	-1.702	0.00854
DARS	-1.661	0.00036
LOX	-1.562	0.00335
YBX3	-1.371	0.00897
RYK	-1.182	0.00256
FREM2	-1.166	0.00953
HDAC6	-1.165	0.00233
CCDC80	-1.080	0.00015
PSMA3	-0.897	0.00137
ANGPT1	-0.874	0.00300
EED	-0.186	0.00930
WNT4	0.768	0.00582
ECM2	0.821	0.00212
REST	0.941	0.00402
SCN3B	0.964	0.00032
FAT3	1.065	0.00760
TIMP3	1.107	0.00346
OLFML1	1.125	0.00085
PTGS2	1.128	0.00540
ZEB1	1.142	0.00373
PRG4	1.167	0.00530
SP1	1.393	0.00735
MMP12	1.400	0.00683
COMP	1.407	0.00111
FREM2	1.420	0.00582
MMP19	1.436	0.00403
SPOCK1	1.444	0.00951
RP11-446E9.1	1.542	0.00992
GYS1	1.612	0.00466
GNAI3	1.625	0.00394
PLXDC1	1.664	0.00964

CX3CL1	1.697	0.00222
FZD3	1.780	0.00848
RAP2B	1.877	0.00024
EPHB4	1.903	0.00017
CDYL	2.174	0.00656
PDGFC	2.181	0.00565
CLEC2B	2.794	0.00338
YWHAZ	3.126	0.00986
IGFL2	3.134	0.00521
IGFL2	3.731	0.00286
ROS1	3.852	0.00019

Supplementary Table S6: Set of compound perturbagens identified from CMap utilizing hits identified from doxorubicin

Description	Score	Name
Anti-inflammatory	34.17	celastrol
ATP synthase inhibitor	37.39	oligomycin-a
ATPase inhibitor	96.34	helveticoside
ATPase inhibitor	93.59	digitoxin
ATPase inhibitor	93.52	cymarin
ATPase inhibitor	92.39	digoxin
ATPase inhibitor	92.34	strophanthidin
ATPase inhibitor	91.59	ouabain
ATPase inhibitor	91.09	digitoxigenin
ATPase inhibitor	88.66	bufalin
ATPase inhibitor	88.11	gitoxigenin
ATPase inhibitor	87.83	proscillaridin
ATPase inhibitor	85.42	cinobufagin
ATPase inhibitor	84.36	oligomycin-c
ATPase inhibitor	57.38	cyclopiazonic-acid
ATPase inhibitor	43.43	thapsigargin
Bile acid	56.75	taurocholic-acid
Bile acid	-92.9	cholic-acid
EGFR inhibitor	-41.57	dovitinib

FGFR inhibitor	-68.52	brivanib
FGFR inhibitor	-85.96	PD-173074
FGFR inhibitor	-91.94	orantinib
FXR antagonist	-95.03	lithocholic-acid
MDM inhibitor	-27.76	MDM2-inhibitor
MDM inhibitor	-73.22	HLI-373
MDM inhibitor	-99.08	RITA
MDM inhibitor	-99.44	SJ-172550
RNA synthesis inhibitor	97.05	daunorubicin
TGF beta receptor inhibitor	-55.9	LY-364947
TGF beta receptor inhibitor	-79.88	D-4476
TGF beta receptor inhibitor	-82.35	SB-525334
TGF beta receptor inhibitor	-92.77	SB-431542
Topoisomerase inhibitor	97.92	pirarubicin
Topoisomerase inhibitor	97.92	pidorubicine
Topoisomerase inhibitor	97.57	doxorubicin
Topoisomerase inhibitor	97.5	mitoxantrone
Topoisomerase inhibitor	91.33	idarubicin
Topoisomerase inhibitor	90.39	amonafide
Topoisomerase inhibitor	87.47	camptothecin
Topoisomerase inhibitor	83.57	topotecan
Topoisomerase inhibitor	76.17	ellipticine
Topoisomerase inhibitor	38.92	etoposide
Topoisomerase inhibitor	-50.62	amsacrine
Topoisomerase inhibitor	-52.97	SN-38
Topoisomerase inhibitor	-55.97	teniposide
Topoisomerase inhibitor	-85.82	irinotecan

Supplementary Table S7: Visualization of synergy scores for Drug Combinations

Drug combination	Synergy score	Most synergistic area score	Method
SB525334 -	13.71	19.38	ZIP

Doxorubicin			
BGJ398 - Doxorubicin	1.49	5.62	ZIP
RITA - Doxorubicin	8.14	14.28	ZIP
SB525334 - Eribulin	5.05	8.93	ZIP
BGJ398 - Eribulin	0.88	5.43	ZIP
RITA - Eribulin	-2.05	1.65	ZIP
SB525334 - Docetaxel	-0.24	2.77	ZIP
BGJ398 - Docetaxel	0.16	2.79	ZIP
RITA - Docetaxel	-1.86	1.39	ZIP
